# Different Dietary Sources of Selenium Alleviate Hepatic Lipid Metabolism Disorder of Heat-Stressed Broilers by Relieving Endoplasmic Reticulum Stress

**DOI:** 10.3390/ijms242015443

**Published:** 2023-10-22

**Authors:** Jiayi Wang, Jinzhong Jing, Zhengyi Gong, Jiayong Tang, Longqiong Wang, Gang Jia, Guangmang Liu, Xiaoling Chen, Gang Tian, Jingyi Cai, Bo Kang, Lianqiang Che, Hua Zhao

**Affiliations:** 1 Key Laboratory for Animal Disease-Resistance Nutrition of Ministry of Education, of China Ministry of Agriculture and Rural Affairs, of Sichuan Province, Animal Nutrition Institute, Sichuan Agricultural University, Chengdu 611130, China; wangjiayi6200@163.com (J.W.); jinzhong0214@163.com (J.J.); hyy121381@163.com (Z.G.); tangjiayong1005@163.com (J.T.); 18380485189@163.com (L.W.); 11988@sicau.edu.cn (G.J.); liugm@sicau.edu.cn (G.L.); xlchen@sicau.edu.cn (X.C.); 13555@sicau.edu.cn (G.T.); 11890@sicau.edu.cn (J.C.); che.lianqiang@sicau.edu.cn (L.C.); 2 College of Animal Science and Technology, Sichuan Agricultural University, Chengdu 611130, China; bokang@sicau.edu.cn

**Keywords:** heat stress, selenium sources, broiler, lipid metabolism disorder, ER stress

## Abstract

As global warming continues, the phenomenon of heat stress (HS) in broilers occurs frequently. The alleviating effect of different selenium (Se) sources on HS-induced hepatic lipid metabolism disorders in broilers remains unclear. This study compared the protective effects of four Se sources (sodium selenite; selenium yeast; selenomethionine; nano-Se) on HS-induced hepatic lipid metabolism disorder and the corresponding response of selenotranscriptome in the liver of broilers. The results showed that HS-induced liver injury and hepatic lipid metabolism disorder, which were reflected in the increased activity of serum alanine aminotransferase (ALT), the increased concentration of triacylglycerol (TG) and total cholesterol (TC), the increased activity of acetyl-CoA carboxylase (ACC), diacylglycerol O-acyltransferase (DGAT) and fatty acid synthase (FAS), and the decreased activity of hepatic lipase (HL) in the liver. The hepatic lipid metabolism disorder was accompanied by the increased mRNA expression of lipid synthesis related-genes, the decreased expression of lipidolysis-related genes, and the increased expression of endoplasmic reticulum (ER) stress biomarkers (PERK, IRE1, ATF6, GRP78). The dietary supplementation of four Se sources exhibited similar protective effects. Four Se sources increased liver Se concentration and promoted the expression of selenotranscriptome and several key selenoproteins, enhanced liver antioxidant capacity and alleviated HS-induced ER stress, and thus resisted the hepatic lipid metabolism disorders of broilers exposed to HS. In conclusion, dietary supplementation of four Se sources (0.3 mg/kg) exhibited similar protective effects on HS-induced hepatic lipid metabolism disorders of broilers, and the protective effect is connected to the relieving of ER stress.

## 1. Introduction

The phenomenon of increased global warming and intensive animal husbandry result in a higher incidence of heat stress (HS) in the breeding industry [1]. In recent years, HS has become a common hazard that impairs animal health and causes significant economic losses to global livestock production [2]. With a high breeding density, rich plumage, lack of sweat glands, and vigorous metabolism, poultry is sensitive to ambient temperatures and more susceptible to HS [3].

As the main metabolic organ of broilers, the liver is a vital hub of lipid metabolism. Unlike mammals, the lipid metabolism of broilers rarely occurs in adipose tissue, with about 70% of fatty acid decomposition occurring in the liver [4]. Hepatic lipid metabolism disorder is a key factor causing fatty liver disease and dyslipidemia [5]. The previous study found that HS causes mitochondrial stress and promotes the production of numerous reactive oxygen species (ROS) during oxidative phosphorylation [6]. Excessive accumulation of ROS impairs redox homeostasis, thus causing liver damage, lipid peroxidation and inflammation [7]. The endoplasmic reticulum (ER) belongs to the membrane system of the cytoplasm and regulates lipid biosynthesis in cells [8]. It is established that HS causes the dysfunction of the ER in hepatocytes [9].

The ER is responsible for the correct folding, assembly, and transport of proteins [8]. Under ER stress, excessive unfolded or misfolded proteins accumulated in the ER lumen activate the unfolded protein response (UPR) [10]. The ER-UPR contributes to improving the folding ability of the ER, which reduces misfolded proteins and accelerates the correct folding of proteins, thus relieving ER stress. The ER-UPR is mainly mediated by three ER stress receptor proteins: inositol-requiring protein 1 (IRE1), activated transcription factor 6 (ATF6), and protein kinase-like ER kinase (PERK) [11]. Under ER stress, ATF6 was transferred to the Golgi and hydrolyzed into the active fragment N-ATF6, which further activates URP [12]. The accumulation of unfolded proteins leads to the self-phosphorylation of IRE1, which cuts the X-box binding protein 1 (XBP1) into the activated XBP-1s, thus promoting UPR [13]. PERK is similar in structure and function to IRE1; the activated PERK leads to eukaryotic translation initiation factor 2α (eIF2α) phosphorylation and inhibits protein translation [14]. However, eIF2α phosphorylation mediates activated transcription factor 4 (ATF4) and promotes the expression of UPR-related genes [15]. Therefore, relieving the ER stress induced by HS may be an effective way to maintain the homeostasis of lipid metabolism.

As an essential trace element for animals, selenium (Se) plays a vital role in antioxidant defense and redox homeostasis maintenance [16]. Selenium performs its biological function mainly through selenoproteins [17]. And, the expression of selenoproteins in organs is effectually regulated by dietary Se supplementation levels [18]. At present, 24 selenoproteins have been identified in poultry [19]. Studies suggest that dietary Se supplementation is involved in hepatic lipid metabolism by regulating the expression of selenoproteins [20,21,22]. Evidence indicates that ER resident selenoproteins contribute to the maintenance of ER homeostasis [23]. Based on the above evidences, we speculate that dietary Se supplementation may alleviate HS-induced hepatic lipid metabolism disorders by promoting the expression of some selenoproteins. Generally, the main supplemental forms of dietary Se in animal production are inorganic Se (sodium selenite, SS), organic Se (selenium yeast, SeY; selenomethionine, SeM), and nano-Se. Due to different transportation and absorption mechanisms, the bioavailability of the different Se sources is different [24,25]. SeM can randomly replace the methionine of proteins in the body, thus forming a Se reservoir, and can be reused when Se intake is insufficient [26]. Nano-Se exhibits novel properties, such as higher biological activity and safety [27]. The liver is the transport and metabolism center of Se. However, whether there are differences among different Se sources in alleviating HS-induced hepatic lipid metabolism disorders in broilers remains unclear. Therefore, our study aims to explore the protective effects of Se on HS-induced hepatic lipid metabolism disorders and compare whether there are differences in the alleviation effects of the four Se sources.

## 2. Results

### 2.1. Liver Weight and Liver Index

**Figure 1:**
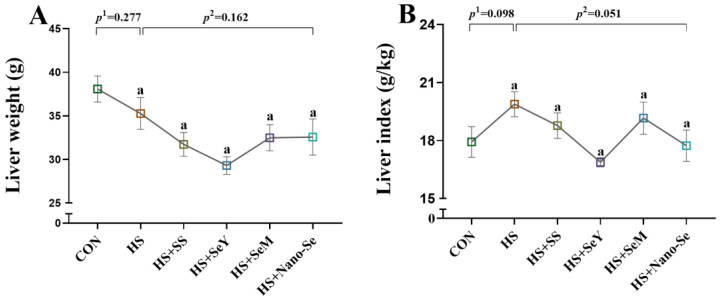
Effects of HS and Se supplementation on the fresh liver phenotypic characteristics of broilers. (**A**) Liver weight; (**B**) Liver index. Results are expressed as the mean ± SE (*n* = 6). The *p* ^1^ value is the *t*-test *p* value between the CON and HS groups, *p* ^2^ is the ANOVA *p* value among the HS groups. Different letters indicate significant difference between each HS groups, with the same letters means there were no significant difference between each HS groups.

We determined the liver weight and liver index of broilers. HS and dietary Se supplementation did not affect liver weight (Figure 1A). Compared with the CON group, HS tended to increase (*p* = 0.098) the liver index, while the dietary supplementation of the four Se sources tended to decrease the liver index of broilers exposed to HS (*p* = 0.051) (Figure 1B).

### 2.2. Serum Biochemical Indicators

As shown in Table 1, HS significantly increased (*p* < 0.05) serum ALT activity, decreased (*p* < 0.05) serum HDL-C and NEFA levels, and tended to decrease the TC (*p* = 0.095) level and increase the TG (*p* = 0.066) level. The four sources of Se showed no impact (*p* > 0.05) on serum ALT, TC, TG and HDL-C levels, while the dietary supplementation of the different Se sources (except SeM) increased (*p* < 0.05) the NEFA level. In addition, the supplementation of the different Se sources (except SS) decreased (*p* < 0.05) serum AST activity. Furthermore, HS or the four sources of Se exhibited limited impact (*p* > 0.05) on serum LDL-C levels.

### 2.3. Se Concentration in Serum and the Liver

**Figure 2:**
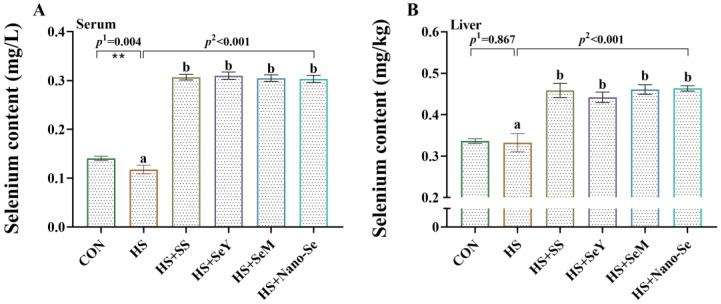
Selenium concentration in serum and the liver. (**A**) Selenium concentration in serum of broilers; (**B**) Selenium concentration in liver of broilers. Results are expressed as the mean ± SE (*n* = 4). The *p* ^1^ value is the *t*-test *p* value between CON and HS group, *p* ^2^ is the ANOVA *p* value among the HS groups. ** means that there is a significant difference between the CON and HS groups, *p* < 0.01. Different letters indicate significant differences between each of the HS groups.

As shown in Figure 2, compared with the CON group, HS decreased (*p* < 0.05) the Se concentration in serum while showing limited impact (*p* > 0.05) on the concentration of Se in the liver. As expected, the dietary supplementation of Se increased (*p* < 0.05) the Se concentration in serum and the liver. Interestingly, no differences were found in the serum and liver Se concentrations (*p* > 0.05) of the broilers in the four Se source groups.

### 2.4. TC Content, TG Content, and HSP70 Protein Abundance in the Liver

**Figure 3:**
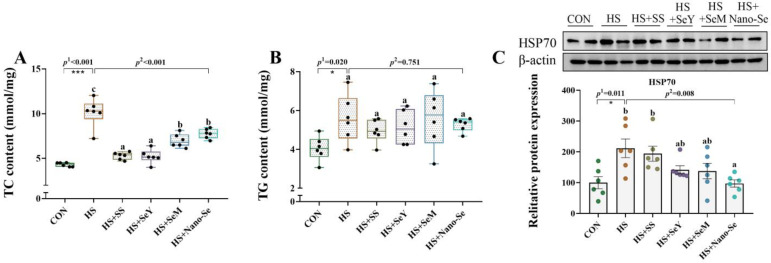
Effects of HS and Se supplementation on the content of TC and TG, and the protein abundance of HSP70 in the livers of broilers. (**A**) TC content; (**B**) TG content; (**C**) Relative protein abundance of HSP70. Results are expressed as mean ± SE (*n* = 6). The *p* ^1^ value is tthe *t*-test *p* value between the CON and HS group, *p* ^2^ is the ANOVA *p* value among the HS groups. “*” means there is a significant difference between the CON and HS groups, * represents *p* < 0.05, *** represents *p* < 0.001. Different letters indicate significant differences between each of the HS groups. The scattered dots represent the measured values of each sample.

We determined the TC content, the TG content, and the protein expression of heat shock protein 70 (HSP70) in the livers of broilers (Figure 3). HS increased (*p* < 0.05) the content of TC (Figure 3A) and TG (Figure 3B), and increased (*p* < 0.05) the protein abundance of HSP70 (Figure 3C) in the livers of broilers. The broilers that received four sources of Se showed lower TC content (*p* < 0.05), while exhibiting a limited impact on the TG content in the liver. In addition, the supplementation of different Se sources (except SS) decreased (*p* < 0.05) or tended to decrease (0.05 < *p* < 0.1) the HSP70 abundance in the liver.

### 2.5. Hepatic Antioxidant Variables

**Figure 4:**
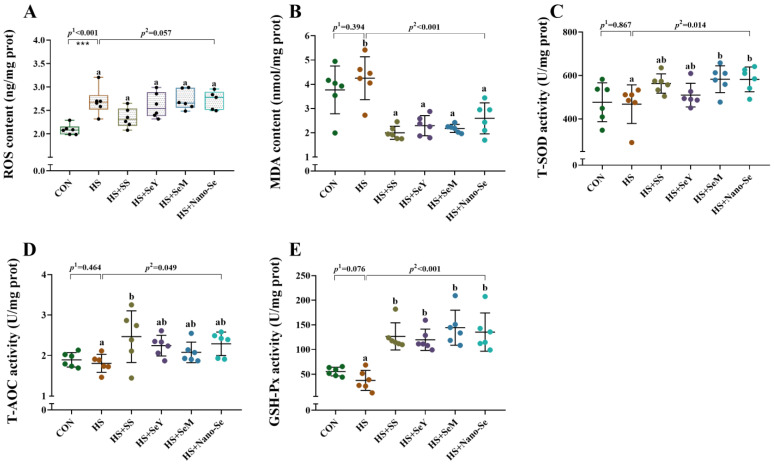
Effects of heat stress and Se supplementation on antioxidant capacity in the livers of broilers. (**A**) ROS content; (**B**) MDA content; (**C**) T-SOD activity; (**D**) T-AOC activity; (**E**) GSH-Px activity. Results are expressed as the mean ± SE (*n* = 6). The *p* ^1^ value is the *t*-test *p* value between the CON and HS groups, *p* ^2^ is the ANOVA *p* value among the HS groups. *** means that there was a significant difference between the CON and HS group, *p* < 0.001. Different letters indicate significant differences between each of the HS groups. The scattered dots represent the measured values of each sample.

As shown in Figure 4, HS increased (*p* < 0.05) the liver ROS level, tended to decrease (*p* = 0.076) the activity of glutathione peroxidase (GSH-Px), while exhibiting a limited impact on (*p* > 0.05) the activity of total superoxide dismutase (T-SOD), total antioxidant capacity (T-AOC), and the content of malondialdehyde (MDA). The dietary supplementation of the four Se sources decreased (*p* < 0.05) the liver MDA concentration, increased (*p* < 0.05) the activity of GSH-Px, and tended to decrease (*p* = 0.067) the liver ROS concentration. In addition, the dietary supplementation of the four Se sources increased (*p* < 0.05) or tended to increase (0.05 < *p* < 0.1) the activity of T-SOD and T-AOC in the liver.

### 2.6. The Activity of Lipid Metabolic-Related Enzymes in the Liver

**Figure 5:**
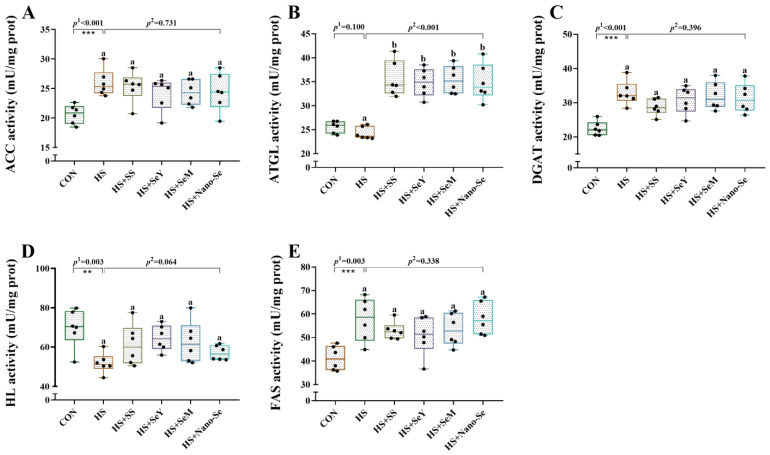
Effects of HS and Se supplementation on lipid metabolism-related enzyme activity in the livers of broilers. (**A**) ACC activity; (**B**) ATGL activity; (**C**) DGAT activity; (**D**) HL activity; (**E**) FAS activity. Results are expressed as mean ± SE (*n* = 6). The *p* ^1^ value is the *t*-test *p* value between the CON and HS groups, *p* ^2^ is the ANOVA *p* value among the HS groups. “*” means that there is a significant difference between the CON and HS groups, ** represents *p* < 0.01, *** represents *p* < 0.001. Different letters indicate significant differences between each of the HS groups. The scattered dots represent the measured values of each sample.

We investigated enzyme activity related to lipid metabolism in the livers of broilers (Figure 5). HS increased (*p* < 0.05) the activity of acetyl-CoA carboxylase (ACC), diacylglycerol O-acyltransferase (DGAT), and fatty acid synthase (FAS) and decreased (*p* < 0.05) the activity of hepatic lipase (HL). Dietary Se supplementation did not affect (*p* > 0.05) the activity of ACC, DGAT, and FAS, but tended to restore (*p* = 0.064) HL activity. Moreover, HS showed limited impact (*p* > 0.05) on the activity of adipose triglyceride lipase (ATGL), while the four sources of Se increased (*p* < 0.05) its activity. However, there were no differences among the four sources of Se.

### 2.7. The Expression of Lipid Metabolic-Related Genes in the Liver

**Figure 6:**
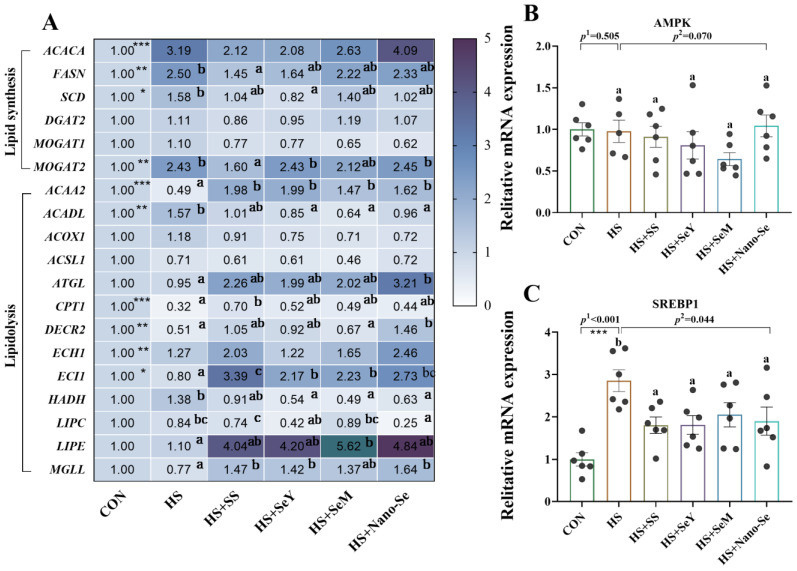
Effects of HS and Se supplementation on the expression of lipid metabolic-related genes in the livers of broilers. (**A**) Expression of lipid metabolic-related genes; (**B**) Expression of AMPK; (**C**) Expression of SREBP1. Results are expressed as the mean ± SE (*n* = 6). The *p* ^1^ value was the *t*-test *p* value between the CON and HS groups; *p* ^2^ is the ANOVA *p* value among the HS groups. “*” means that there is a significant difference between the CON and HS groups, * represents *p* < 0.05, ** represents *p* < 0.01, *** represents *p* < 0.001. Different letters indicate significant differences between each of the HS groups. The scattered dots represent the measured values of each sample.

We further investigated the mRNA profiles of lipid metabolism-related genes in the livers of broilers (Figure 6). For lipid synthesis-related genes (Figure 6A), HS increased (*p* < 0.05) the expression of acetyl-CoA carboxylase alpha (*ACACA*), fatty acid synthetase (*FASN*), monoacylglycerol O-acyltransferase 2 (*MOGAT2*) and stearoyl-CoA desaturase (*SCD*) at mRNA levels. Compared with the HS group, Se supplementation did not affect the expression of *ACACA*, while additional Se decreased (*p* < 0.05) or exhibited a limited impact on (0.05 < *p* < 0.1) the mRNA abundance of *FASN*, *SCD*, and *MOGAT2*. HS and dietary Se supplementation exhibited no impact (*p* > 0.05) on the mRNA levels of diacylglycerol transferase 2 (*DGAT2*) and monoacylglycerol O-acyltransferase 1 (*MOGAT1*).

Heat stress and Se supplementation also affected the expression of lipolysis-related genes (Figure 6A). HS decreased (*p* < 0.05) the mRNA expression of acetyl Coa acyltransferase 2 (*ACAA2*), carnitine palmitoyltransferase 1 (*CPT1*), 4-dienoyl-CoA reductase 2 (*DECR2*), and enoyl-CoA delta isomerase 1 (*ECI1*). However, HS increased the mRNA expression of acyl-CoA long-chain dehydrogenase (*ACADL*) and noyl-CoA hydratase 1 (*ECH1*), while exhibiting no impact (*p* > 0.05) on the expression of other lipolysis-related genes. The dietary supplementation of the four Se sources generally increased the expression of these lipolysis-related genes, except for *ACADL*, acetyl-Coa oxidase 1 (*ACOX1*), long-chain lipoyl CoA synthetase (*ACSL1*), *ECH1*, *HADH*, and *LIPC*.

We also investigated the signals that regulate lipid metabolism. HS exerted no impact (*p* > 0.05) on the mRNA expression of Adenosine monophosphate (AMP)-activated protein kinase (*AMPK*) (Figure 6B), while the addition of the four Se sources tended to (*p* = 0.070) decrease its expression. In addition, HS increased (*p* < 0.05) the mRNA expression of sterol regulatory element binding protein 1 (*SREBP1*) (Figure 6C) while the addition of the four Se sources decreased (*p* < 0.05) its expression.

### 2.8. ER Stress Biomarkers in the Liver

**Figure 7:**
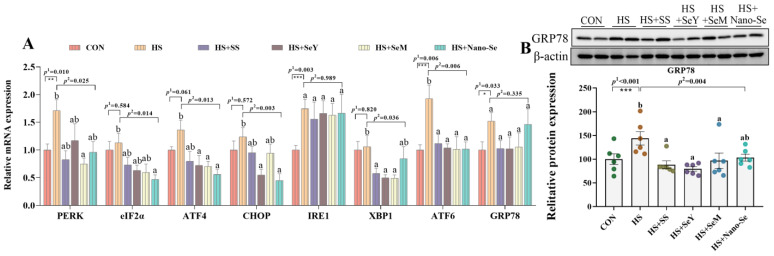
Effects of HS and Se supplementation on the ER stress biomarkers in the liver of broilers. (**A**) The mRNA abundance of ER stress biomarkers; (**B**) Relative protein abundance of GRP78. Results are expressed as the mean ± SE (*n* = 6). The *p* ^1^ value is the *t*-test *p* value between the CON and HS groups; *p* ^2^ is the ANOVA *p* value among the HS groups. “*” means that there was a significant difference between the CON and HS groups, * represents *p* < 0.05, ** represents *p* < 0.01, *** represents *p* < 0.001. Different letters indicate significant differences between each of the HS groups. The scattered dots represent the measured values of each sample.

As shown in Figure 7, relative to the CON group, HS increased (*p* < 0.05) the mRNA abundance of *PERK*, *IRE1*, *ATF6*, and Glucose-Regulated Protein 78 (*GRP78*), and tended to increase (*p* = 0.061) the mRNA level of *ATF4* in the livers of broilers (Figure 7A). The dietary supplementation of the four Se sources decreased (*p* < 0.05) the mRNA expression of *PERK*, *ATF4*, and *ATF6*, and exerted no impact (*p* > 0.05) on the mRNA expression of *IRE1* and *GRP78*. Meanwhile, although HS did not affect the mRNA levels of *eIF2α*, C/EBP homologous protein (*CHOP*), and *XBP1*, Se supplementation lowered (*p* < 0.05) their mRNA expression. We further explored the protein expression of GRP78 (Figure 7B). Heat stress increased (*p* < 0.05) the protein abundance of GRP78 while the dietary supplementation of the four Se sources resulted in a recovery in the protein abundance of GRP78. However, there were no significant differences among the four sources of Se on the regulation of ER stress biomarkers.

### 2.9. The mRNA Expression of Selenotranscriptome in the Liver

**Figure 8:**
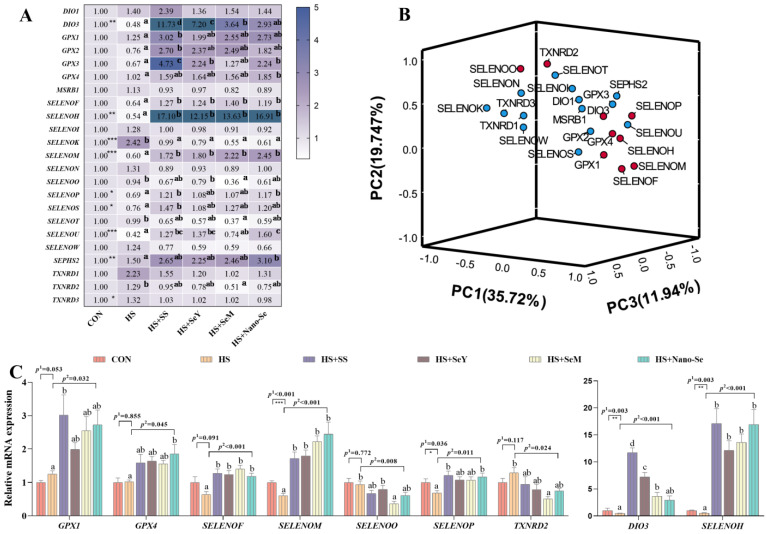
Effects of HS and Se supplementation on the expression of selenotranscriptome and key selenogenes in the liver. (**A**) Heat map of mRNA expression of the selenotranscriptome; (**B**) Principal component analysis for the determination of key selenogenes; (**C**) Relative mRNA expression of these key selenogenes. Results are expressed as the mean ± SE (*n* = 6). The *p* ^1^ value is the *t*-test *p* value between the CON and HS groups; *p* ^2^ is the ANOVA *p* value among the HS groups. “*” means that there was a significant difference between the CON and HS groups, * represents *p* < 0.05, ** represents *p* < 0.01, *** represents *p* < 0.001. Different letters indicate significant differences between each of the HS groups.

Selenium exerts its biological function mainly through selenoproteins. Therefore, we investigated the mRNA expression of selenotranscriptome in the livers of broilers. As shown in Figure 8A, HS downregulated (*p* < 0.05) the mRNA levels of *DIO3*, *SELENOH*, *SELENOM*, *SELENOP*, *SELENOS*, and *SELENOU*, while the four sources of Se increased the mRNA expression of these selenogenes. However, HS upregulated (*p* < 0.05) the expression of *SELENOK*, *SEPHS2*, and *TXNRD3* while the supplementation of the four Se sources downregulated (*p* < 0.05) the expression of *SELENOK* and *SEPHS2* but exerted no impact (*p* > 0.05) on the expression of *TXNRD3*. Although HS did not affect (*p* > 0.05) the mRNA expression of *GPX1*, *GPX2*, *GPX3*, *GPX4*, *SELENOF*, *SELENOO*, *SELENOT*, and *TXNRD2*, Se supplementation increased (*p* < 0.05) the expression of *GPX1*, *GPX2*, *GPX3*, *GPX4*, and *SELENOF* and decreased (*p* < 0.05) the expression of *SELENOO*, *SELENOT*, and *TXNRD2*. While HS and the different sources of Se did not affect (*p* > 0.05) the remaining six selenogenes, the four Se sources showed a similar restorative effect on the expression of these selenogenes under HS conditions.

We further performed a principal component analysis to determine the key selenogenes regulated by HS and Se supplementation. In the present study, nine of the twenty-three selenogenes were located relatively far away in the three-dimensional space. Therefore these nine selenogenes (*GPX1*, *GPX4*, *DIO3*, *SELENOF*, *SELENOH*, *SELENOM*, *SELENOO*, *SELENOP*, and *TXNRD2*) were key selenogenes. Among them, selenoprotein F and selenoprotein M encoded by *SELENOF* and *SELENOM* were ER resident proteins. Except for *TXNRD2* and *SELENOO*, the mRNA expressions of the other seven selenogenes were upregulated by the additional four Se sources compared with the HS group (Figure 8C).

### 2.10. Correlation Analysis

**Figure 9:**
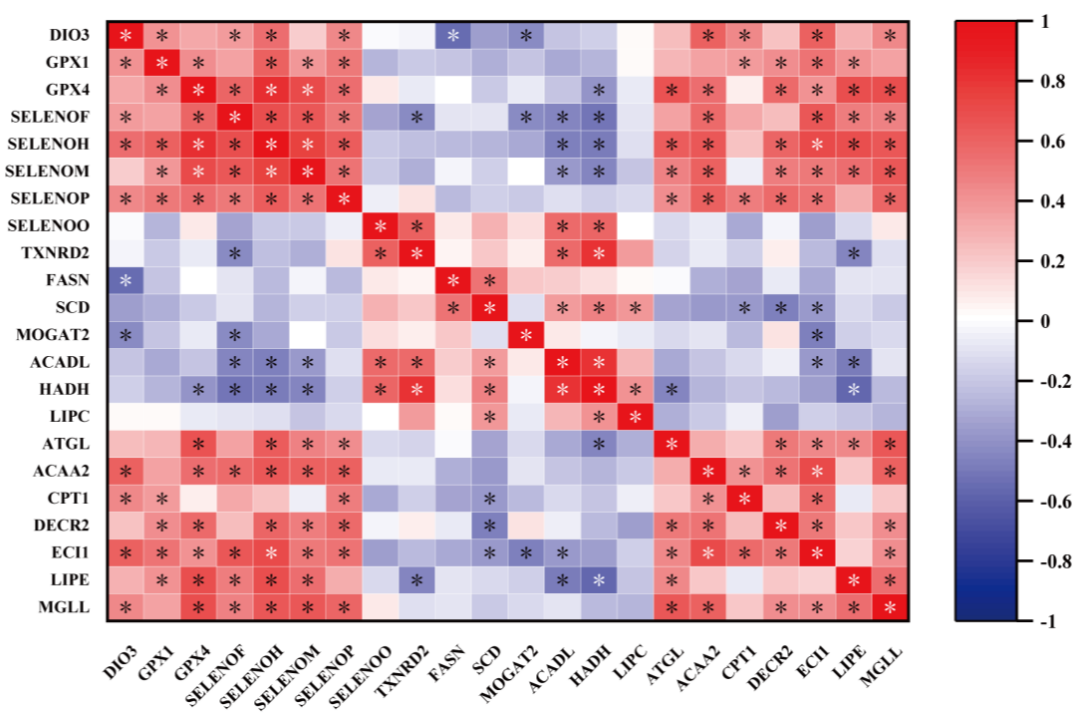
Correlation analysis for key selenogenes and lipid metabolic-related genes. “*” with white or black indicates that there is a significant correlation (*p* < 0.05). The color red represents a positive correlation and the color blue represents a negative correlation.

Here, we performed a correlation analysis to determine the correlation between key selenogenes and lipid metabolism (Figure 9). For lipid synthesis, *FASN* was negatively correlated with *DIO3* (*p* < 0.05). *MOGAT2* was negatively correlated with *DIO3* and *SELENOF* (*p* < 0.05). For lipolysis, *ACADL* and *HADH* were negatively correlated with *SELENOH*, *SELENOF*, and *SELENOM* (*p* < 0.05) and positively correlated with *SELENOO* and *TXNRD2* (*p* < 0.05). *ATGL* and *DECR2* were positively correlated with four selenogenes (*GPX4*, *SELENOH*, *SELENOM* and *SELENOP*) (*p* < 0.05), and DECR2 was also positively correlated with *GPX1* (*p* < 0.05). *ACAA2*, *ECI1*, and *MGLL* were positively correlated with six selenogenes (*GPX4*, *DIO3*, *SELNEOF*, *SELENOH*, *SELENOM*, and *SELENOP*) (*p* < 0.05). *CPT1* was positively correlated with three selenogenes (*GPX1*, *DIO3*, and *SELENOP*) (*p* < 0.05). Diacylglycerol lipase (*LIPE*) was positively correlated with five selenogenes (*GPX1*, *GPX4*, *SELENOF*, *SELENOH*, and *SELENOM*) (*p* < 0.05) and negatively correlated with *TXNRD2* (*p* < 0.05).

### 2.11. The Protein Abundance of Selenoproteins

**Figure 10:**
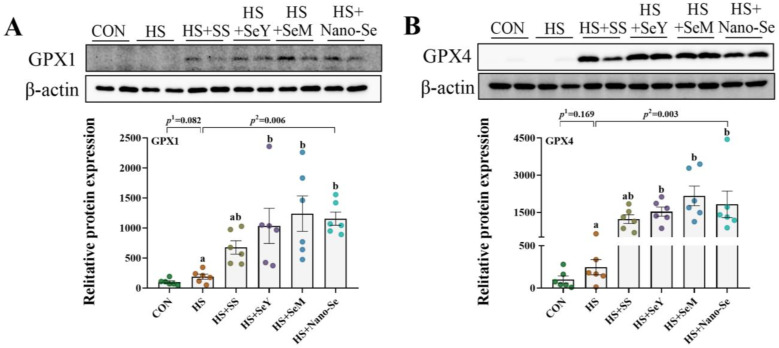

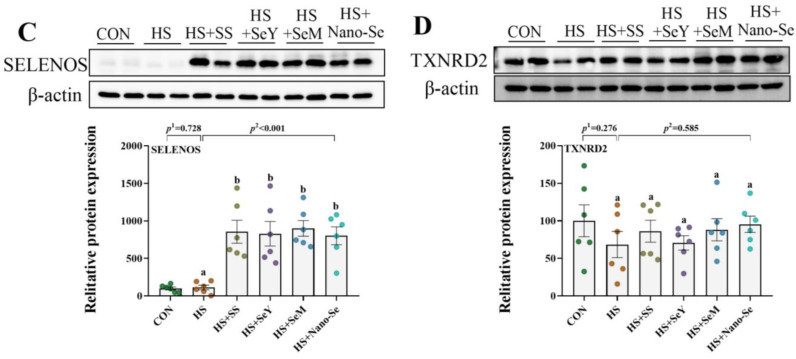
Effects of HS and Se supplementation on the protein expression of four selenoproteins. (**A**) Protein expression of GPX1 in liver; (**B**) Protein expression of GPX4 in liver; (**C**) Protein expression of SELENOS in liver; (**D**) Protein expression of TXNRD2 in liver. Results are expressed as the mean ± SE (*n* = 6). *The p* ^1^ value is the *t*-test *p* value between the CON and HS groups; *p* ^2^ is the ANOVA *p* value among the HS groups. Different letters indicate significant differences between each of the HS groups. The scattered dots represent the measured values of each sample.

As shown in Figure 10, we further validated the expression of four key selenogenes (*GPX1*, *GPX4*, *SELENOS*, and *TXNRD2*) at the protein level. HS showed limited impact (*p* > 0.05) on the protein expression of GPX1, GPX4, and SELENOS, but tended to increase the protein expression of GPX1. Except for dietary SS which showed a limited impact on (0.05 < *p* < 0.1) the protein expression of GPX1 and GPX4, the supplementation of the other three Se sources promoted (*p* < 0.05) the protein expression of GPX1, GPX4 and SELENOS. At the same time, Se supplementation exhibited limited impact (*p* > 0.05) on the protein expression of TXNRD2.

## 3. Discussion

As the key organ for broilers, liver is the hub of fatty acid synthesis and lipid circulation [28]. Heat stress caused by prolonged hyperthermia causes liver damage and impairs lipid metabolism in animals [22]. In the present study, HS decreased serum HDL-C and NEFA levels and increased serum ATL activity. The content of HDL-C and NEFA can reflect the lipid metabolism status in the body. HS decreased the serum NEFA level, which is consistent with a previous study in mammals [29]. HDL-C removes cholesterol from tissues and blood and transports it to the liver for transformation and elimination [30]. The increased activity of ALT and AST in serum is generally accompanied by liver injury and metabolic disorders [31,32]. Our results in the present study confirm that HS causes liver damage and affects lipid metabolism in broilers. Current evidence suggests that Se supplementation contributes to the improvement of lipid metabolism disorders in the livers and skeletal muscle of animals under stress conditions [33,34]. In our study, the supplementation of four Se sources restored serum HDL-C and NEFA concentrations, and decreased serum AST activity to a certain extent, which implies the alleviating effects of Se on liver lipid metabolism disorders.

In the present study, HS tended to increase the liver index. Heat-stressed animals cannot fully mobilize fat for energy supplementation, and thus show higher fat deposition [35]. Therefore, the increased concentration of TG and TC in the livers of broilers exposed to HS may be due to abnormal lipid metabolism. A previous study reported that Se supplementation affects liver lipid metabolism and decreases cholesterol concentration in the livers of broilers [20]. In our study, all of the supplemented four Se sources consistently reduced liver TC levels, indicating that Se supplementation contributes to the improvement of lipid metabolism disorders in the livers of broilers exposed to HS.

Heat shock proteins (HSPs) are molecular chaperones, which contribute to and promote the proper folding of proteins [36]. In addition, heat shock protein 70 (HSP70) is generally used as a biomarker to evaluate HS damage in livestock [37]. In this study, HS upregulated the protein expression of HSP70, and the addition of Se inhibited the expression of HSP70, except for SS. This result suggests that the regulation ability of SS on HSP70 is lower than that of other Se sources. However, the specific mechanisms underlying the disparities require further study.

The damage caused by HS is mainly mediated by oxidative stress, which is manifested as increased ROS levels and decreased antioxidant capacity [38]. A previous study revealed that Se alleviates HS damage by scavenging harmful free radicals [39]. In the present study, HS elevated the liver ROS level of broilers. As an excellent antioxidant, dietary Se supplementation can effectively improve the antioxidant capacity of the liver [33]. As expected, the supplementation of four Se sources improved the antioxidant capacity of the livers in broilers exposed to HS, which was mainly reflected in the increased activity of GSH-Px and T-SOD and the decreased concentration of MDA. In addition, the different Se sources showed similar improvements in the antioxidant capacity of the liver.

Previous studies indicated that HS enhances lipid biosynthesis and suppresses lipidolysis in the livers of broilers by regulating the activity of enzymes related to lipid metabolism [33,40]. In this study, HS increased the activity of ACC, DGAT, and FAS and decreased the activity of HL in the liver. FAS, DGAT, and ACC are enzymes related to lipid synthesis. For fatty acid synthesis, ACC catalyzes the synthesis of malonyl CoA, while FAS is a critical rate-limiting enzyme in the lipid synthesis process [41]. As the only rate-limiting enzyme in TG synthesis, DGAT catalyzes the esterification reaction of diacylglycerol (DAG) and fatty acyl-CoA to form TG [42]. HL is mainly distributed in the liver and catalyzes chylomicrons and promotes the hydrolysis of TG in very low-density lipoproteins [43]. The decreased activity of HL corresponds to lipid catabolism inhibited by HS. The above results were consistent with increased TG content in the liver, which further proved that HS causes hepatic lipid metabolism disorder. The four sources of Se all increased the activity of ATGL while showing no impact on the activity of the other four enzymes related to lipid metabolism. ATGL is a key enzyme in the process of lipolysis that specifically hydrolyzes the first ester bond of TG [44]. These results suggest that Se may inhibit fat deposition caused by HS by enhancing the activity of enzymes related to lipolysis.

We further examined the mRNA expression of lipid metabolism-related genes. For lipid synthesis-related genes, HS upregulated the expressions of *ACACA*, *SCD*, *FASN*, *MOGAT2*, and *SREBP1* in the liver. *ACACA* is a key gene for the de novo synthesis of fatty acids [45]. As a vital nuclear transcription factor, SREBP1 regulates the expression of downstream factors ACC, FAS, and SCD and promotes lipid synthesis [46]. *FASN* encodes the enzyme FAS, which promotes the process of fatty acid chain extension [47]. SCD exists in the ER and catalyzes the formation of saturated fatty acids into monounsaturated fatty acids, thus promoting the synthesis of TG and cholesterol [48]. MOGAT2 mediates a key step in TG synthesis in the liver, which catalyzes the esterification of glycerol 1 to glycerol 2 [49]. However, the effects of the different Se sources on these lipid synthesis-related genes were generally consistent; they decreased or tended to decrease the expression of the above lipid synthesis-related genes. For lipolysis-related genes, HS downregulated the expression of *CPT1*, *ACAA2*, *ECI1*, and *DECR2* and upregulated the expression of *ACADL* and *ECH1*. Among those genes, CPT1 is located in the mitochondrial intima and acts as a carrier during the oxidation of fatty acid β [50]. ECH1 and ACAA2 are located in the mitochondria and participate in fatty acid β-oxidation [51]. ECI1 converts trans- or cis-3-allyl CoA to trans-2-allyl CoA for further oxidation [52]. As rate-limiting enzymes, DECR2 mediates the oxidative decomposition of polyunsaturated fatty acids and ACADL mediates the β-oxidation of long-chain fatty acids [53,54]. A previous study reported that dietary Se supplementation alleviates hepatic injuries, reduces adipocyte size, and decreases the expression of lipid synthesis-related genes in high-cholesterol-diet-fed rats [55]. Similarly, in the present study, the supplementation of the four Se sources increased the expression of most lipolysis-related genes. The above results suggest that HS causes hepatic lipid metabolism disorder by inhibiting lipid decomposition and promoting lipid synthesis. Se supplementation promotes the mRNA expression of lipid decomposition genes and suppresses the expression of lipid synthesis-related genes, thus contributing to liver lipid metabolism homeostasis under HS.

As an important place for lipid synthesis in cells, the ER is closely related to lipid metabolism. ER stress promotes lipid synthesis in the livers of broilers [33]. To relieve ER stress, cells produce the UPR though three sensors: IRE1, PERK, and ATF6 [10,11]. And, several downstream factors such as eIF2α, ATF4, CHOP, and XBP1 participate in the alleviation of ER stress. The current study showed that HS increased the mRNA expressions of *PERK*, *eIF2α*, *ATF4*, *CHOP*, and *ATF6* and increased the protein expression of GRP78, while the four sources of dietary Se lowered the expression of these ER stress biomarkers. GRP78 is a main regulator of UPR; it acts as a molecular chaperone to promote the proper folding of proteins to relieve ER stress [56]. Our present study confirmed that HS induces ER stress and that the dietary supplementation of the four Se sources alleviates hepatic ER stress in broilers. The four sources of Se shared similar ameliorating effects.

Selenium performs its biological function mainly through selenoproteins [17]. Selenoproteins perform multiple biological functions such as antioxidant and metabolic stabilization functions [22]. Our previous studies suggest that Se supplementation can effectively promote the protein expression of selenoproteins in the organs of animals [33,34,57]. Therefore, we further investigated the effects of HS and the supplementation of the four Se sources on the mRNA expression of selenotranscriptome in the liver. Firstly, HS decreased the mRNA abundance of six selenogenes (*DIO3*, *SELENOH*, *SELEOM*, *SELENOP*, *SELENOS*, and *SELENOU*). *SELENOM* overexpression inhibits lipid accumulation and shows a protective effect on nonalcoholic fatty liver disease [58]. SELENOS is a core component of the retrotranslocation channel in ER-associated protein degradation, which contributes to the protein folding process [59]. over the overexpression of SELENOH protects cell integrity by reducing cell ROS production and restoring mitochondrial homeostasis [60]. SELENOU is a nonmammalian selenoprotein that is widely expressed in all tissues and shows a redox function [61]. Therefore, the downregulation of the above selenogenes may reflect the liver oxidative stress and lipid metabolism disorder induced by HS. Secondly, the expression of *SELENOK*, *SEPHS2*, and *TXNRD3* was upregulated under HS, which may indicate their important roles in protecting cells against damage caused by HS. SEPHS2 is essential for the biosynthesis of selenoproteins [62]. A previous study reports that the disruption of ER homeostasis led to the overexpression of SELENOK, which binds misfolded proteins and transports them to ER-associated degradation [63]. The supplementation of the four Se sources increased the expression of 12 selenogenes, which indicates that Se exerts its anti-HS effects by promoting the expression of selenoproteins.

Principal component analysis of the selenotranscriptome was performed to identify the selenoproteins that play key roles in the alleviation of ER stress and the regulation of lipid metabolism. Nine selenogenes (*GPX1*, *GPX4*, *DIO3*, *SELENOF*, *SELENOM*, *SELENOO*, *SELENOP*, and *TXNRD2*) were identified as key selenogenes. Selenoproteins such as GPX1, GPX4, SELENOO and TXNRD2 are important parts of the body’s antioxidant system and participate in redox homeostasis regulation [64,65,66,67]. In addition, SELENOF and SELENOM are ER resident selenoproteins. SELENOF is involved in ER glycoprotein folding and quality control [59]. SELENOM is an ER-resident oxidoreductase, which improves antioxidant capacity [68]. It has been reported that SELENOF and SELENOM can act as cofactors of protein disulfide isomerase and regulate ER homeostasis [69]. These ER-resident selenoproteins contribute to protein folding and ER homeostasis regulation [23]. We further verified four key selenoproteins at protein levels. HS exhibited a limited impact on the protein abundance of GPX1, GPX4, SELENOS, and TXNNRD2. The supplementation of the four Se sources increased the protein expression of GPX1, GPX4, and SELENOS. Based on these results, dietary Se supplementation contributes to maintaining lipid metabolism homeostasis and relieving ER stress by regulating the expression of selenotranscriptome and key selenoproteins, and limited differences were found among the four Se sources.

Selenoproteins are involved in lipid metabolism [33]. Thus, we evaluated the correlation between lipid metabolism-related genes and key selenogenes. Most of the key selenogenes were positively correlated with lipid catabolism genes. These results are consistent with the previous studies where the upregulation of selenogenes affected the expression of lipidolysis-related genes [21,33,70]. Our present results suggest that selenoproteins regulate lipid metabolism homeostasis by regulating the expression and activity of lipid metabolism-related enzymes.

In the present study, the dietary supplementation of the different Se sources increased Se concentrations in serum and the liver, while no differences were found among the four Se source groups. This is consistent with a previous study where the dietary supplementation of 0.3 mg/kg of Se from different sources showed the same Se levels in plasma and the liver [71]. The liver is the main metabolic organ for Se metabolism and reservoir, and birds under HS shared similar liver Se levels among the four Se source groups. This may partly explain that the dietary supplementation of the four Se sources exhibited similar alleviation effects on the HS-induced hepatic lipid metabolism disorders of broilers. Some differences between the Se groups were found, such as the protein expression of GPX1 and the mRNA expression of DIO3, etc. These differences may be due to the different efficiency of the conversion of different Se sources to selenoproteins. In addition, Se concentrations were the total level of Se in the liver or serum, including unmetabolized Se, Se from selenoproteins, and Se from proteins that contain Se. However, the differences in individual indicators did not affect the overall protective effect of the different Se sources.

## 4. Materials and Methods

### 4.1. Animal, Diet, and Experimental Design

The Animal Care and Use Committee of Sichuan Agricultural University approved this animal trial (SCAUAC202107-2). SS, SeY, SeM and nano-Se for animal feeding were obtained from Chengdu Shuxing Feed Co., Ltd. (Sodium selenite type II, contain 0.45% Se, Chengdu, China), Angel Yeast Co., Ltd. (FUBON 2000 ppm, contain 0.2% Se, Chengdu, China), Sichuan Sinyiml Biotechnology Co., Ltd. (L-selenomethionine type II, contain 0.2% Se, Mianyang, China) and Sichuan Chelota Biotech Co., Ltd. (Nano-Se, contain 0.3% Se, Guanghan, China), respectively.

A total of 480 arbor acre broilers with the similar average body weight of 650 ± 50 g (aged 21 days) were allotted into 6 dietary treatments with 8 replicates of 10 broilers per replicate. During the experiment, the control group (CON) and HS group were fed a basal diet under a thermoneutral (22 ± 2 °C) or hyperthermal environment (33 ± 2 °C), respectively. The following four treatment groups were fed a basal diet supplemented with 0.3 mg/kg Se in the form of SS (HS + SS), SeY (HS + SeY), SeM (HS + SeM), and nano-Se (HS + nano-Se) under hyperthermal conditions (33 ± 2 °C). The actual analyzed Se concentration in the diets is shown in Supplementary Figure S1. The nutritional requirements were adequate according to NRC, 1994 and NY/T33-2004 (Supplementary Table S1) [72,73]. All broilers had free access to water and their diet and housed in a temperature-controlled coop as required. Temperature and relative humidity were continuously monitored on a daily basis for the entire experimental period.

### 4.2. Liver Weight and Sample Collection

A total of 36 broilers (six broilers per group) were selected on day 22. To avoid body weight differences affecting the results, we weighed these eight replicates separately in each treatment group and removed the maximum and minimum replicate according to the average body weight. Then, in the remaining six replicates of each group, six broilers with a body weight close to the average litter weight were selected for sample collection. After overnight fasting, blood samples were collected from the internal jugular vein using a sterile vacuum tube and stored in sterile centrifuge tubes at –20 °C after centrifuging (3000× *g*) for 10 min for subsequent analyses. After the broilers were slaughtered, all livers were weighed and recorded as liver weight. The liver indexes were estimated as follows: liver index (g/kg) = liver weight/broiler’s live weight. The collected liver samples were rapidly frozen in liquid nitrogen and then stored at –80 °C for further analysis.

### 4.3. Serum Biochemical Analyses

The activity of ALT, AST, and ALP in the serum of the broilers as well as the concentration of TG, TC, LDL-C, HDL-C, and NEFA in the serum of the broilers were determined by using a biochemistry analyzer (3100, HITACHI, Tokyo, Japan).

### 4.4. Selenium Concentration in Diets, Serum and Liver

An atomic fluorescence spectrometer (AFS-3100, Hai Guang Instrument, Beijing, China) was used to determine the total Se concentrations, including the diets, serum, and liver. The experimental method and sample digestion procedure were according to the national standard of China (GB 5009.93-2010) [74].

### 4.5. Liver Biochemical Analyses

We used corresponding assay kits (no. A005-1-2, A001-1-2, A015-1-2, A003-1-2, A100-1-1, A111-1-1, Nanjing Jiancheng Bioengineering Institute, Nanjing, China) to determine the activity of GSH-Px, T-SOD, and T-AOC and the concentrations of MDA, TG, and TC in the livers of the broilers in each treatment group. In addition, we used enzyme-linked immunosorbent assay (ELISA) kits (no. 3430401, 150401, 6064101, 6012001, 202701, 6019701, Jiangsu Meimian Industrial Co., Ltd., Yancheng, China) to analyze the enzyme activity of ACC, FAS, DGAT, HL, and ATGL as well as the concentration of ROS in the livers of the broilers. And, we used a bicinchoninic acid (BCA) protein assay kit (no. AR0197, Nanjing Jiancheng Bioengineering Institute, Nanjing, China) to determine the total protein levels of each sample. An enzyme-labeling instrument (Model 680, Bio-Rad, Hercules, CA, USA) was used to determine the final optical density values.

### 4.6. Q-PCR Analyses of mRNA Abundance

The relative mRNA expression of all target genes was determined using the Q-PCR method in accordance with a previous study [33]. In brief, the isolation and reverse transcription of total RNA in the liver samples was performed using RNAiso Plus and PrimeScript RT reagent kits (no. 9109, RR047A, Takara, Dalian, China). The amplification of the target genes was determined using the SYBR Premix Ex TaqTM II kit (no. A402-01, Exongen, Chengdu, China) by a QuantStudio 6 Flex system (Applied Biosystems, Waltham, MA, USA). The primer sequences used for 21 lipid metabolic-related genes, 8 ER stress markers, 23 selenogenes, and 1 reference gene (β-Actin) were picked from the National Center for Biotechnology Information and listed in Supplementary Table S2. Finally, the 2^−∆∆Ct^ method was used to normalize the relative mRNA expression [22].

### 4.7. Western Blot Analyses

The Western blot was used to determine the expression of all target proteins, and the process was performed in accordance with a previous study [75]. The primary antibodies used in present study were as follows: GRP78 (1:1000), GPX1 (1:1000), GPX4 (1:2000), and TXNRD2 (1:1000) (200310-4F11, 616958, 513309, R26013; Zen BioScience, Chengdu, China); HSP70 (1:5000) (ab5439; Abcam, Cambridge, UK); SELENOS (1:1000) (15591-1-AP, ProteinTech Group, Chicago, IL, USA); and β-Actin (1:5000; MAB1501; Millipore, Darmstadt, Germany).

### 4.8. Statistical Analysis

Statistical analysis was performed using SPSS 27.0 software (SPSS Inc., Chicago, IL, USA). The CON and HS groups were compared using independent samples *t*-tests. The treatment groups (HS, HS + SS, HS + SeY, HS + SeM and HS + nano-Se) were analyzed using one-way ANOVA, followed by Duncan’s multiple range tests. Results are expressed as means with their standard errors (SEM). The significance level was accepted at *p* < 0.05, and 0.05 ≤ *p* < 0.1 was assumed to be a trend. Principal component analysis was performed using SPSS 27.0 (SPSS, Inc., Chicago, IL, USA). Correlation analysis was performed using Origin 2021 (OriginLab, Northampton, MA, USA).

## 5. Conclusions

In conclusion, HS caused hepatic lipid metabolism disorder in livers of broilers by inducing ER stress. Se supplementation in four different forms (SS, SeY, SeM and nano-Se) increased the Se concentration in the liver and moderately improved the antioxidant capacity and relieved ER stress, thus alleviating the hepatic lipid metabolism disorder of broilers. The protective effects of Se are generally achieved by regulating the expression of selenotranscriptome and key selenoproteins. Correlation analysis revealed that these key selenoproteins can regulate lipid metabolic homeostasis by affecting the expression of a few key lipid metabolism-related enzymes. Though the expression of individual genes or proteins among different Se source groups is inconsistent, the four sources of Se supplementation have similar protective effects on the hepatic lipid metabolism disorder of broilers. Therefore, at the dose of 0.3 mg/kg, the four sources of selenium all exhibited a protective effect on the HS-induced hepatic lipid metabolism disorders of broilers.

## Figures and Tables

**Table 1:** Effects of HS and Se supplementation on the serum biochemical indicators of broilers.

Item	Groups						SEM	*p*-Value ^1^	*p*-Value ^2^
	CON	HS	HS + SS	HS + SeY	HS + SeM	HS + Nano-Se			
ALT/(U/L)	11.05 *	15.46	13.80	12.71	12.30	13.68	0.77	0.019	0.775
AST/(U/L)	336.04	365.50 ^b^	286.45 ^ab^	283.33 ^a^	283.97 ^a^	276.19 ^a^	10.25	0.310	0.016
TC/(mmol/L)	3.14	2.68	2.66	2.78	2.82	2.80	0.10	0.095	0.983
TG/(mmol/L)	0.42	0.95	0.75	0.82	0.74	0.98	0.60	0.066	0.338
HDL-C/(mmol/L)	2.28 **	1.50	1.69	1.68	1.76	1.68	0.06	0.007	0.807
LDL-C/(mmol/L)	0.45	0.44	0.34	0.36	0.28	0.43	0.03	0.937	0.198
NEFA/(mmol/L)	0.78 ***	0.31 ^a^	0.51 ^b^	0.50 ^b^	0.42 ^ab^	0.47 ^b^	0.02	<0.001	0.026

ALT, alanine aminotransferase; AST, aspartate aminotransferase; TC, total cholesterol; TG, triacylglycerol; HDL-C, high density lipoprotein cholesterin; LDL-C, low density lipoprotein cholesterin; NEFA, non-esterified fatty acid. Results are expressed as the mean ± SE (*n* = 6). The *p* ^1^ value is the *t*-test *p* value between the CON and HS group; *p* ^2^ is the ANOVA *p* value among the HS groups. “*” means that there is a significant difference between the CON and HS groups, * represents *p* < 0.05, ** represents *p* < 0.01, *** represents *p* < 0.001. Different letters indicate significant differences between each of the HS groups.

## Data Availability

Data described in the manuscript will be made available upon request pending application and approval.

## Supplementary Materials

The supporting information can be downloaded at: https://www.mdpi.com/article/10.3390/ijms242015443/s1.
