# Supplementary material for: Different Dietary Sources of Selenium Alleviate Hepatic Lipid Metabolism Disorder of Heat-Stressed Broilers by Relieving Endoplasmic Reticulum Stress
Source: Int J Mol Sci. 2023 Oct 22;24(20):15443. doi: 10.3390/ijms242015443 (PMC10607182; doi:10.3390/ijms242015443)
Supplement: Supplementary file 1 [file ijms-24-15443-s001.zip › Supporting Material.pdf]

Supplementary Table S1. Composition and nutrient levels of the basal diet (air-dry basis, %)

Ingredients	Phase	
	0-3W	4-6W
Corn	58.50	63.14
Soybean meal	33.42	31.20
Fish meal	1.80	-
Soybean oil	2.35	2.00
$CaCO_3$	1.40	1.25
$CaHPO_4$	1.45	1.58
NaCl	0.40	0.35
Lys-HCl	0.04	0.02
DL-Met	0.26	0.16
L-Thr	0.03	-
Choline chloride	0.15	0.10
Premix[1]	0.20	
Premix[2]		0.20
Total	100.00	100.00
Nutrient composition		
ME (MJ/kg)	12.27	12.33
CP, %	20.89	19.01
Ca, %	1.01	0.90
TP, %	0.68	0.65
AP, %	0.41	0.38
Lys, %	1.15	1.00
Met, %	0.58	0.45
Met+Cys, %	0.91	0.76
Thr, %	0.82	0.72
Val, %	0.96	0.87

Trp, %	0.24	0.22

Note: (1) Premix[1] provides the following per kg of the diet: Viamin A, 8000 IU; Viamin D_3, 2000 IU; Viamin E, 20 IU; Viamin K_3, 3.2 mg; Viamin B_1, 2 mg; Viamin B_2, 6.4 mg; Viamin B_6, 4 mg; Viamin B_{12}, 0.2 mg; D-biotin, 1.1 mg; D-pantothenic acid, 12 mg; Nicotinamide, 40 mg; Folic acid, 1 mg; Fe (FeSO4·7H2O), 100 mg; Cu (CuSO4·5H2O), 8 mg; Mn (MnSO4·H2O), 120 mg; Zn (ZnSO4·H2O), 100 mg; I (KI), 0.7 mg

(2) Premix[2] provides the following per kg of the diet: Vitamin A, 6000 IU; Vitamin D_3, 1500 IU; Vitamin E, 15 IU; Vitamin K_3, 2.4 mg; Vitamin B_1, 1.5 mg; Vitamin B_2, 4.8 mg; Vitamin B_6, 3 mg; Vitamin B_{12}, 0.15 mg; D-biotin, 0.83 mg; D-pantothenic acid, 9 mg; Nicotinamide, 30 mg; Folic acid, 0.75 mg; Fe (FeSO4·7H2O), 80 mg; Cu (CuSO4·5H2O), 8 mg; Mn (MnSO4·H2O), 100 mg; Zn (ZnSO4·H2O), 80 mg; I (KI), 0.7 mg

Supplementary Table S2. Primers used for the Q-PCR

Gene	Accession number	Primer pairs (5′ to 3′ direction)
**Housekeeping control genes**		
β-Actin	AY550069	F:CCCAAAGCCAACCGTGAGAA<br>R:CCACGTACATGGCTGGGGTG
**Lipid metabolic-related genes**		
ACACA	NM_205505.2	F:CATGTCTGGTTTGCACCTCGTA<br>R:CCCGCTGCACGTCAACTTT
FASN	NM_205155.4	F:CATTGCACGGAGTCAAACTAGTTATC<br>R:TCCAGACGGCAGATTTCAGTAAG
SCD	NM_204890.2	F:GCGCTGCTCACATGTTTGG<br>R: TCTCCCGTGGGTTGATGTTC
DGAT2	XM_040661934.2	F:CCGTGAACCGTGACAGCATA<br>R:CCACGATGATGATGGCATTG
MOGAT1	XM_015277034.4	F:GGGAAGCCCCATCCTTGTAAA<br>R:GCTGCTCAACCTCTTCATTGGT
MOGAT2	XM_040661931.2	F:GGAACAGAGGCAACAGGCTTT<br>R:GAAACCATAAGGAGAGCATCATCAG
SREBP1	NM_204126.3	F:AGTCGGCGATCCTGAGGAA<br>R:TCCTTGAGGGACTTGCTCTTCTG
AMPK	NM_001039603.2	F:CGGCGGCAGATAAACAGAAG<br>R:GCCCAGAATGTAATGCCCAAT
ACSL1	NM_001012578.2	F:CGCACCCTTCCGACAAATAC<br>R:GGTCGTGAGGGCTGCAAAA
ATGL	EU240627.2	F:ATCACGGTGTCTCCGTTCTCA<br>R:CACGCATGTTTGTGGAACTGTCT
LIPE	XM_040695200.2	F:GAGGGCTTTGCTGTGTTTGG<br>R:AACCCGAGACACCTCCCATA

MGLL	NM_001277143.2	F:ACTCAACCTCGTGCTTCCAAAC<br>R:CGGGAAATTGCACTTGGATCT
CPT1	XM_046918285.1	F:AAGCAAAAACCAGCCCTGATG<br>R:CCCATGTCTCGGTAGTGAGCAA
ACADL	NM_001006511.3	F:GGGAGGAGCAGATGTACGTCAA<br>R:GGGCATGACTATATCGGAATGG
ECHS1	NM_001277395.2	F:ATTGGCTGCGCTGAGAAGATC<br>R:AGCAGCATTGACGGACTCCTT
HADH	NM_001277897.2	F:GCAAATCACGCAGTTGGCTAA<br>R:GAAGAAATGGAGGCCACCAAA
ACAA2	NM_001006571.3	F:CATGAGCCAGGCGCCTTAT<br>R:AGTTCTGCTCCTAATCTGGTTCCA
LIPC	XM_046899314.1	F:CATGTTTCAGGGTTTGCTGGTAGT<br>R:TCAAGGCCTGTAATTCTTCCAATC
ACOX1	NM_001006205.2	F:GGATTTTTTGCAGGCGGGTAT<br>R:CACACGCTGGTTCACCTGAGT
ECI1	NM_001277585.2	F:CAGGCGTAGCCACGATGAA<br>R:AGTGAGGAAGTCCAGGCTGAGA
DECR2	XM_040683253.2	F:GGCCGAGGCCTCAAAAAAG<br>R:TTTGCGGCTGCCTGACAT
**Endoplasmic reticulum stress biomarkers**		
PERK	XM_040671515.2	F:GTGGCTGACTGGAAGGTTATGG<br>R:CAATTGGAGTGCAAAACTGGTATTC
eIF2α	NM_001031323.2	F:AAACCTGCGCAAGCGTATAGAC<br>R:CCTCATGAAGCTTGGAACTTGTTAC
ATF4	NM_204880.3	F:CCACACGTTACCGGCAAAA<br>R:AAATCTCTGCACTCCCCTGACA
CHOP	KP064314.1	F:TGCCGTGCTTAGCAGAATGG<br>R:ATGCTGTACAGTGGTGCTGGAA
IRE1	NM_001285499.2	F:GGCTGTGTCTTTTATTATGTGGTATCTG<br>R:TGGCTTGTCGCTGTAGAGATTTG
XBP-1	NM_001006192.2	F:CCATTCCTGACAACCTCCACAT<br>R:CGAACAGGAGATCAGACTCAGAATC
ATF6	XM_040677276.2	F:ATGCAGAGCTCCGTTAGTCAGAA<br>R:CTGAGGCTCGTGTCTGTGTAAGG
GRP78	NM_205491.2	F:CGGACGATGAGGAGAAAAAGG<br>R:GAATAGGTGGTACCGAGGTCGAT
**Selenoprotein-encoding genes**		
DIO1	NM_001324555.3	F:TGCGCGCGGTCAAACTT<br>R:TTGCCCTTGGCTATGTGGATT
DIO3	NM_001122648.3	F:CAGTACAAAACCCGGCTCCA<br>R:TACACTTGGATGACCACCGC

GPX1	NM_001277853.3	F:ACCATGTTCGAGAAGTGCGA<br>R:TCTCTCAGGAAGGCGAACAG
GPX2	NM_001277854.3	F:GGTCCTCATCGAGAACGTGG<br>R:GCTGGGTGTAATCCCTCACC
GPX3	NM_001163232.3	F:CACCATCTACGACTACGGGG<br>R:TCTTCCCCGCGTACTTTCTG
GPX4	NM_001346449.2	F:GTGATGCTCCCCTTCGTCTC<br>R:TACAGGTAGGCGGGCAGAT
MSRB1	NM_001135558.3	F:CCCGCAGGAGAATTAAGCGA<br>R:GCTCCCATACACCCTGACTG
SELENOF	NM_001012926.3	F:AGTACGTGCGTGGTTCTGAC<br>R:GCAATGTTCCCACTGTCGTC
SELENOH	NM_001277865.2	F:GCCGTAGAGATCAACCCGC<br>R:GACACCTCGAAGCTGTTCCT
SELENOI	NM_001031528.4	F:TGCCAGCCTCTGAACTGGAT<br>R:TGCAAACCCAGACATCACCAT
SELENOK	NM_001025441.2	F:ATAAATCACTGGGGTGGAGGC<br>R:GCTCCTTTGCCTGCTTCTTAC
SELENOM	NM_001277859.2	F:ACATCCCGCTGTACCATAACCT<br>R:TCTCCTCCCGGGTCATGTC
SELENON	NM_001114972.3	F:CTGTATGGGGCGAGTGAAGG<br>R:TATCACAGAGGGGACCGAGG
SELENOO	NM_001115017.5	F:CCCAGCGTTAACCGGAATGA<br>R:TCTTGCCGTCCGCTTTGTTA
SELENOP	NM_001031609.3	F:CCAAGTGGTCAGCATTCACATC<br>R:ATGACGACCACCCTCACGAT
SELENOS	NM_001024734.3	F:CCGACATGGTGGTAAGAAGACA<br>R:GCTTGTGCATTCAACTCCTCTTG
SELENOT	NM_001006557.4	F:GATCTGCGTCTCCTGAGGTT<br>R:GTAGTTCTCCCCCTCGATGC
SELENOU	NM_001193519.3	F:GGCTGCTTCGGAAATGTCT<br>R:CTGTTATGGCTGCGCCAAC
SELENOW	NM_001166327.2	F:TGTGGGTCTGCTTTACGCC<br>R:AAGCTGGAAGGTGCAAAATGAA
SEPHS2	NM_001366334.2	F:AGGTTGACGAGTTACAGCGA<br>R:CTCCGGCACTTTACAGCCTC
TXNRD1	NM_001030762.4	F:GCCTTACTGTCCGGGGAAAA<br>R:CCTGCACATTCCAAGGCAAC
TXNRD2	NM_001122691.3	F:AAAGATGCCCAGCACTACGG<br>R:GCTTGAGCCATCACAGACCA
TXNRD3	NM_001122777.3	F:TGACCTCTTCTCCCTGCCTTA<br>R:AAGCACCCACAACTAGCGTT
