# Supplementary figures and images for: Different Dietary Sources of Selenium Alleviate Hepatic Lipid Metabolism Disorder of Heat-Stressed Broilers by Relieving Endoplasmic Reticulum Stress
Source: Int J Mol Sci. 2023 Oct 22;24(20):15443. doi: 10.3390/ijms242015443 (PMC10607182; doi:10.3390/ijms242015443)

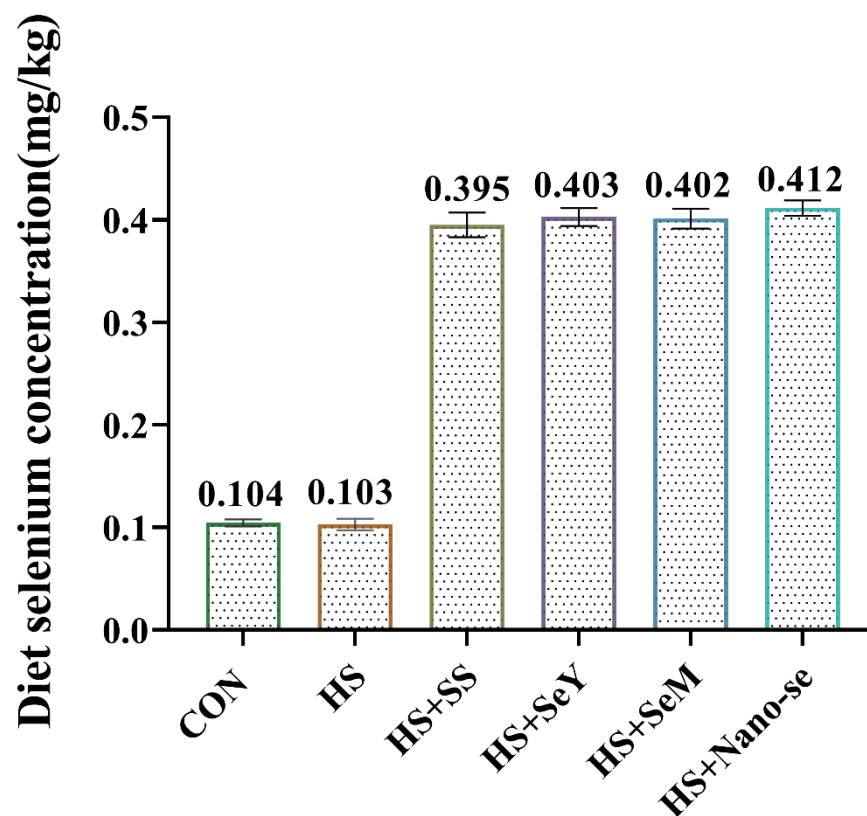

**Supplementary Figure S1.** Selenium concentration in diets. Results were expressed as mean  $\pm$  SE ( $n = 4$ ).

Supplement: Supplementary file 1 [file ijms-24-15443-s001.zip › Caption explaining for Supplementary Figure 1.pdf]
